# 

**Published:** 1863-11

**Authors:** 


### DENTAL REGISTER

### OF THE WEST.

EDITED BY

J. TAFT AND GEO. WATT.

all NOVEMBER.—1863.

**MONTHLY:**
**Three Dollars per Annum, in advance.**

CINCINNATI:
J. TAFT, PUBLISHER.

J. Taft, Dentist, 172 Race Street.

